# The availability, spatial accessibility, service utilisation and retrieval cost of paediatric intensive care services for children in rural, regional and remote Queensland: study protocol

**DOI:** 10.1186/1472-6963-13-163

**Published:** 2013-05-02

**Authors:** Lauren M Thompson, Nigel R Armfield, Anthony Slater, Christian Mattke, Michele Foster, Anthony C Smith

**Affiliations:** 1 Centre for Online Health, School of Medicine, The University of Queensland, Brisbane, Australia; 2 Queensland Children’s Medical Research Institute, The University of Queensland, Brisbane, Australia; 3 Paediatric Intensive Care Unit, Royal Children’s Hospital, Brisbane, Australia; 4 Paediatric Intensive Care Unit, Mater Children’s Hospital, Brisbane, Australia; 5 School of Social Work and Human Services, The University of Queensland, Brisbane, Australia

**Keywords:** Paediatrics, Paediatric intensive care, Retrieval, Regionalisation, Health service organisation, Spatial accessibility

## Abstract

**Background:**

Specialist health services are often organised on a regionalised basis whereby clinical resources and expertise are concentrated in areas of high population. Through a high volume caseload, regionalised facilities may provide improved clinical outcomes for patients. In some cases, regionalisation may be the only economically viable way to organise specialist care. While regionalisation may have benefits, it may also disadvantage some population groups, particularly in circumstances where distance and time are impediments to access.

Queensland is a large Australian state with a distributed population. Providing equitable access to specialist healthcare services to the population is challenging. Specialist care for critically ill or injured children is provided by the Queensland Paediatric Intensive Care Service which comprises two tertiary paediatric intensive care units. The two units are located 6 km (3.7 miles) apart by road in the state capital of Brisbane and provide state-wide telephone advice and specialist retrieval services. Services also extend into the northern area of the adjacent state of New South Wales. In some cases children may be managed locally in adult intensive care units in regional hospitals.

The aim of this study is to describe the effect of geography and service organisation for children who need intensive care services but who present outside of metropolitan centres in Queensland.

**Methods/design:**

Using health services and population data, the availability and spatial accessibility to paediatric intensive care services will be analysed. Retrieval utilisation and the associated costs to the health service will be analysed to provide an indication of service utilisation by non-metropolitan patients.

**Discussion:**

While the regionalisation or centralisation of specialist services is recognised as an economical way to provide specialist health services, the extent to which these models serve critically ill children who live some distance from tertiary care has not been described. This study will provide new information on the effect of the regionalisation of specialist healthcare for critically ill children in Queensland and will have relevance to other regionalised health services. This study, which is focussed on describing the organisation, supply and demands on the health service, will provide the foundation for future work to explore clinical outcomes for non-metropolitan children who require intensive care.

## Background

In many health systems, specialist services are organised on a regionalised basis whereby large facilities are located in areas of high population.

In some specialities [1-4], it has been shown that regionalisation may offer improved patient outcomes by concentrating clinical resources and expertise, and by the range of clinical problems and professional development opportunities that higher caseloads present. Economically, regionalisation may also be a cost-effective way to provide specialist public health services [5].

While regionalisation may have benefits, it may also disadvantage sectors of the population who live some distance from care. Queensland is a large state with an area of greater than 1.7 million square kilometres and a population of 4.5 million. While the majority of the state’s population live in the south east corner and in large regional centres along the east coast, a significant number of people live inland in large centres, smaller towns and isolated communities [6].

Queensland’s public health system has a responsibility to provide an appropriate level of care for its residents regardless of their geographic location. Because many specialist health services are regionalised to larger centres, meeting this responsibility presents significant challenges. In some cases, health care services have been centralised, for instance care for critically ill children, the subject of this work, is provided by a central service (the Queensland Paediatric Intensive Care Service). Under this arrangement, services are provided by two paediatric intensive care units (PICUs) at the Royal Children’s Hospital (RCH) and the Mater Children’s Hospital (MCH) located within 6 km (3.7 miles) of each other in Brisbane. Where necessary, emergency retrieval services are used to transport children to an appropriate level of care using road ambulance, helicopter or fixed-wing aircraft, with the mode of transport being determined by distance, terrain and by the child’s condition.

For families in regional or remote areas, the centralisation of PICU services can result in distance and time impediments to urgently needed health care. In some cases, children from these areas may be managed in regional centres closer to their home where the population may be sufficient to support a sophisticated general intensive care unit (ICU) [6]. Such local management has advantages: it avoids the risks associated with transport; it reduces dislocation from family and community; and it relieves pressure on PICU services [7]. However, there may also be disadvantages: research suggests that children have better outcomes when treated in paediatric focused facilities rather than general intensive care services [8,9].

To the authors’ knowledge, the effect of geography on health care for critically ill children in Queensland has not been described. Thus, this study aims to provide new information on service availability, spatial accessibility, service utilisation and retrieval cost for children who require intensive care services, but who present away from a tertiary facility.

## Methods/design

### Design

The study will use health service and population data to describe the availability, spatial accessibility, service utilisation and retrieval cost of providing paediatric intensive care services for patients who present outside of metropolitan areas in Queensland.

In some cases, children are managed in adult ICUs in regional hospitals. When calculating supply ratios, because our aim is to describe the availability of paediatric intensive care services, only facilities with a designated PICU will be included. However, for spatial and cost analysis, because children in north Queensland are retrieved to the ICU at The Townsville Hospital (TTH), paediatric retrievals to that hospital will also be included in the analysis to gain an understanding of distance and time impediments and retrieval costs in that region.

Geographic analyses will be conducted using ArcGIS (Esri, New York, USA). Statistical analyses will be conducted using Stata (Stata Corp, College Station, USA). All analyses will be conducted using health service data for the period 2009 to 2011 and Australian Bureau of Statistics (ABS) population projections for 2010 from 2006 based data [10].

The study has been approved by the Queensland Children’s Health Services (RCH) Human Research Ethics Committee (HREC/11/QRCH/176) and The University of Queensland Medical Research Ethics Committee (2012000131).

### Service availability

Supply ratios, expressed as the number of PICU beds per 1,000 child population for the state will be calculated. Bed numbers will be based on 2012 health service data.

### Spatial accessibility

Three studies will be conducted to describe the distance and time to paediatric intensive care services for children in Queensland.

The first study aims to describe accessibility to PICU services from each place of possible demand in the state. The Statistical Local Area (SLA) will be used as the geographical unit of analysis, with the centroid of each SLA being used as a proxy for location of potential demand. Euclidean distances will be calculated between each SLA centroid and the mid-point between the two Brisbane PICUs (place of supply). Child population and distance results will be summarised for each SLA. Subsequently, the cumulative proportion of the child population stratified in 100 km (62 miles) steps from the PICUs will be calculated using population data.

A second study will use retrospective paediatric retrieval service data as a proxy for the utilisation of paediatric intensive care services by children residing outside of the metropolitan area. Distance and time to care from patient’s usual residence to the PICU will be calculated using appropriate measures for the usual mode of transport (network analysis for locations usually serviced by road ambulance and the combination of both road network analysis and Euclidean distance for areas usually served by fixed-wing or helicopter). Distance and round-trip time from point of retrieval to paediatric intensive care services (RCH and MCH PICUs) will be calculated. For air retrievals, Euclidean distance will be calculated in nautical miles; for road ambulance the most direct road route will be calculated in kilometres. For time, total mission duration will be extracted from retrieval records and the mean, range and standard deviation of mission times will be described.

Using the same method as the second study, a third study will use retrospective paediatric retrieval data for the TTH adult ICU to calculate distance and time to care for children retrieved to that unit in North Queensland.

### Cost from the health service perspective

Retrospective data on retrieval duration, transport mode and staffing will be obtained from the records of each PICU and from the Townsville ICU. Staffing costs will be calculated using hourly Queensland Health wage rates for the relevant year and mission duration. Road ambulance, helicopter and fixed-wing aircraft transport costs will be obtained from the relevant service providers. The cost of flight and road ambulance crew will be assumed to be included in the overall transport costs unless otherwise specified. Costs will be calculated for each tertiary centre and for all centres combined. An average retrieval cost will be calculated by dividing the total cost of paediatric retrievals by the number of children retrieved in the study period. All costs will be reported in Australian Dollars.

## Discussion

The study will provide new information on the availability, spatial accessibility, retrieval utilisation and cost of paediatric intensive care services for children in Queensland, a state, where unique demographic and geographic conditions prevail. The results of the study may be useful for future health-service service planning and will provide a foundation for future work to explore clinical outcomes for non-metropolitan children who require intensive care.

## Abbreviations

ABS: Australian Bureau of Statistics; ICU: Intensive Care Unit; MCH: Mater Children’s Hospital; RCH: Royal Children’s Hospital; PICU: Paediatric Intensive Care Unit; TTH: The Townsville Hospital.

## Competing interests

The authors declare that they have no competing interests.

## Authors’ contributions

All authors have contributed to the development of the study. LMT conceived the study in consultation with NRA, MF and ACS. LMT designed the study and drafted the manuscript. AS and CM contributed to and refined the design. All authors contributed to refinement of the manuscript and have read and approved the final version.

## Pre-publication history

The pre-publication history for this paper can be accessed here:

http://www.biomedcentral.com/1472-6963/13/163/prepub
